# Classification of High Intensity Zones of the Lumbar Spine and Their Association with Other Spinal MRI Phenotypes: The Wakayama Spine Study

**DOI:** 10.1371/journal.pone.0160111

**Published:** 2016-09-20

**Authors:** Masatoshi Teraguchi, Dino Samartzis, Hiroshi Hashizume, Hiroshi Yamada, Shigeyuki Muraki, Hiroyuki Oka, Jason Pui Yin Cheung, Ryohei Kagotani, Hiroki Iwahashi, Sakae Tanaka, Hiroshi Kawaguchi, Kozo Nakamura, Toru Akune, Kenneth Man-Chee Cheung, Noriko Yoshimura, Munehito Yoshida

**Affiliations:** 1 Department of Orthopaedic Surgery, Wakayama Medical University, 811-1 Kimiidera, Wakayama, Japan, 641–8509; 2 Department of Orthopaedics and Traumatology, The University of Hong Kong, Professorial Block, 5th Floor 102 Pokfulam Road, Pokfulam, Hong Kong, SAR, China; 3 Department of Joint Disease Research, 22nd Century Medical & Research Center, Faculty of Medicine, The University of Tokyo, 7-3-1 Hongo, Bunkyo-ku, Tokyo, Japan, 113-8655; 4 Department of Medical Research and Management for Musculoskeletal Pain, 22nd Century Medical & Research Center, Faculty of Medicine, The University of Tokyo, 7-3-1 Hongo, Bunkyo-ku, Tokyo, Japan, 113-8655; 5 Department of Orthopaedic surgery, Faculty of Medicine, The University of Tokyo, 7-3-1 Hongo, Bunkyoku, Tokyo, 113–8655, Japan; 6 Japan Community Healthcare Organization Tokyo Shinjuku Medical Center, 5–1 Tsukudo-chome, Shinjuku-ku, Tokyo, Japan, 162–8543; 7 Rehabilitation Services Bureau, National Rehabilitation Center for Persons with Disabilities, 1 Namiki 4-chome, Tokorozawa City, Saitama, Japan, 359–8555; Kanazawa University, JAPAN

## Abstract

**Introduction:**

High intensity zones (HIZ) of the lumbar spine are a phenotype of the intervertebral disc noted on MRI whose clinical relevance has been debated. Traditionally, T2-weighted (T2W) magnetic resonance imaging (MRI) has been utilized to identify HIZ of lumbar discs. However, controversy exists with regards to HIZ morphology, topography, and association with other MRI spinal phenotypes. Moreover, classification of HIZ has not been thoroughly defined in the past and the use of additional imaging parameters (e.g. T1W MRI) to assist in defining this phenotype has not been addressed.

**Materials and Methods:**

A cross-sectional study of 814 (69.8% females) subjects with mean age of 63.6 years from a homogenous Japanese population was performed. T2W and T1W sagittal 1.5T MRI was obtained on all subjects to assess HIZ from L1-S1. We created a morphological and topographical HIZ classification based on disc level, shape type (round, fissure, vertical, rim, and enlarged), location within the disc (posterior, anterior), and signal type on T1W MRI (low, high and iso intensity) in comparison to the typical high intensity on T2W MRI.

**Results:**

HIZ was noted in 38.0% of subjects. Of these, the prevalence of posterior, anterior, and both posterior/anterior HIZ in the overall lumbar spine were 47.3%, 42.4%, and 10.4%, respectively. Posterior HIZ was most common, occurring at L4/5 (32.5%) and L5/S1 (47.0%), whereas anterior HIZ was most common at L3/4 (41.8%). T1W iso-intensity type of HIZ was most prevalent (71.8%), followed by T1W high-intensity (21.4%) and T1W low-intensity (6.8%). Of all discs, round types were most prevalent (anterior: 3.6%, posterior: 3.7%) followed by vertical type (posterior: 1.6%). At all affected levels, there was a significant association between HIZ and disc degeneration, disc bulge/protrusion and Modic type II (p<0.01). Posterior HIZ and T1W high-intensity type of HIZ were significantly associated with disc bulge/protrusion and disc degeneration (p<0.01). In addition, posterior HIZ was significantly associated with Modic type II and III. T1W low-intensity type of HIZ was significantly associated with Modic type II.

**Conclusions:**

This is the first large-scale study reporting a novel classification scheme of HIZ of the lumbar spine. This study is the first that has utilized T2W and T1W MRIs in differentiating HIZ sub-phenotypes. Specific HIZ sub-phenotypes were found to be more associated with specific MRI degenerative changes. With a more detailed description of the HIZ phenotype, this scheme can be standardized for future clinical and research initiatives.

## Introduction

Since the advent of magnetic resonance imaging (MRI), there has been a tremendous interest to identify unique spinal phenotypes (e.g. patterns of intervertebral disc degeneration (DD), Modic changes, endplate abnormalities) that may be representative of the degenerative disc process and that may provide insight into determining the painful disc level(s) [1–7]. High-intensity zones (HIZ) of the lumbar spine are an example of a disc phenotype that have gathered widespread interest since their initial report in 1992 by Aprill and Bogduk [8]. Based on their report, HIZ was described as a hyperintense signal in the posterior annulus fibrosus of the disc on T2-weighted (T2W) MRI using only a relatively low-strength 0.6 Tesla scanner in patients with low back pain (LBP) undergoing discography. Since then, numerous reports have surfaced attempting to address the clinical relevance of HIZ and its relationship with LBP, but the significance of this association remains under heated debate [8–16]. Some studies have suggested that lumbar HIZ is related to a concordant pain response on discography and have concluded it to be a significant MRI biomarker for the diagnosis of LBP [8–11]. Alternatively, others studies have not found any association between HIZ with LBP [12–16]. To further complicate this issue, the prevalence of HIZ in symptomatic and asymptomatic populations has varied greatly between reported studies [8–16]. Besides symptomatology, additional controversies exist with regards to its pathology, natural history, and morphology/topography [1, 8–16]. This may be attributed to the lack of a strict phenotype definition of HIZ, proper sampling of the study samples with appropriate demographics, standardized imaging assessment methods, insufficient statistical analyses and consideration of occupational/lifestyle factors, limited knowledge regarding its relationship with other spinal phenotypes, and the poor imaging resolution of particular MRI sequences [1, 8–16].

Understanding the pathogenesis of HIZ is necessary to clearly define its clinical significance with regards to LBP. Previous reports suggested that HIZ was an effect of annular tears leading to an accumulation of disc material that is toxic to the disc and surrounding neural structures, and may cause further degenerative changes within the intervertebral disc [9, 10, 13, 17, 18]. Alternatively, annular tears were also reported to appear in the early stages of DD [19]. Therefore, the relationship between HIZ and DD remains unclear. Traditionally, annular tears require discography, an invasive examination, in order to determine the type of tear that produces degenerative changes and pain. The MRI is a non-invasive method used to characterize HIZ but there is currently no standardized classification system for researchers to phenotype HIZ and most descriptions are based solely upon T2W MRI. As such, these concerns need to be addressed since they are an important initial step to better understand the pathobiology, prevalence, etiology, and clinical significance of HIZ. In addition to the lack of standardized phenotyping, the role of varying morphological/topographical traits of HIZ remains unknown and demand attention.

Coupling of T2W and T1W MRI sequences have been found useful to elaborate upon various spinal phenotypes, such as Modic changes and their classification, and have shed light upon their clinical relevance and decision-making [1, 20–27]. However, to date, no such approach has been adopted for HIZ. Therefore, utilizing a multimodal MRI approach to better characterize the HIZ phenotype is imperative to assist communication between study centers and aid large scale cross-cohort and cross-ethnic analyses. Furthermore, better understanding of HIZ may contribute to more sensitive identification of symptomatic disc levels, prediction and progression of disc or adjacent endplate changes, and potential use for patient selection for regenerative therapies for the disc. It also has potential to be a marker for identifying patients at risk for adjacent segment degeneration/disease in relation to a fusion or arthroplasty procedure.

Due to the limitations as addressed, better classification and understanding of HIZ is needed. Thus, this current study’s objectives are three-fold and are based on a large-scale, population-based study. Firstly, we aimed to address the prevalence and morphological/topographical variations of HIZ throughout the lumbar spine using both T2W and T1W MRI. This imaging mapping further facilitated the creation of a novel classification of HIZ. Secondly, we aimed to assess the association of HIZ with other MRI spinal phenotypes.

## Methods

### Participants

This was a cross-sectional study based on the *Wakayama Spine Study* [28–34], a large population-based study created to address the etiology of common spinal disorders in Japan. Our study population was a sub-cohort of the large-scale population-based cohort study called *Research on Osteoarthritis/Osteoporosis Against Disability* (ROAD). The ROAD study was a nationwide, prospective study of bone and joint diseases consisting of population-based cohorts established in three communities in Japan [35–38]. The participants of ROAD study were recruited from listings of resident registrations in three communities that have different characteristics based on three geographical regions: an urban region in “I town” (Tokyo); a mountainous region in “H town” (Wakayama); and a coastal region in “T town” (Wakayama). *The Wakayama Spine Study* started in mountainous region H town and coastal region T town in Wakayama from 2008 as a population-based cohort [28–34]. For the current study, recruited subjects were 20 years of age or older, irrespective of gender residing in T town who were willing to respond to a survey distributed in 2013.

The inclusion criteria were the ability to walk to the survey site, report data, and sign an informed consent form. Subjects with spinal tumors, infections, chronic inflammatory conditions, previous posterior spinal fusion operation, contraindicated to MRI (e.g., pacemakers) and/or other disqualifiers (e.g., pregnant) were excluded. In total, 857 individuals underwent MRI of the lumbar spine. However, 43 MRI results were not available due to incomplete T1W and T2W sagittal lumbar spine MRI or of quality too poor to read for HIZ. The *Wakayama Spine Study* obtained approval from the local ethics committee of the University of Tokyo, the Tokyo Metropolitan Institute of Gerontology, and Wakayama Medical University. All participants provided their own written informed consent.

### MRI Assessment

Lumbar MRI were performed using a mobile MRI unit (Achieva 1.5 T; Philips Medical Systems, Best, The Netherlands) for all participants. On the same day of imaging assessment, participants also completed standardized questionnaires and underwent anthropometric examination, which accounted for height (meters) and weight (kilograms) as well as additional subject demographics (e.g. age [years], sex-type). All participants underwent MRI in the supine position. The imaging protocol included sagittal T2W fast-spin echo (FSE), with a repetition time (TR) of 3000 ms/echo and an echo time (TE) of 120 ms. The field of view (FOV) was 270 × 270 mm. The sagittal T1W FSE was with a TR of 540 ms/echo, a TE of 10 ms and a FOV of 270 × 270 mm. All cuts were 5mm thick and 11 total slices were available.

### Evaluation of MRI

**Fig 1:**
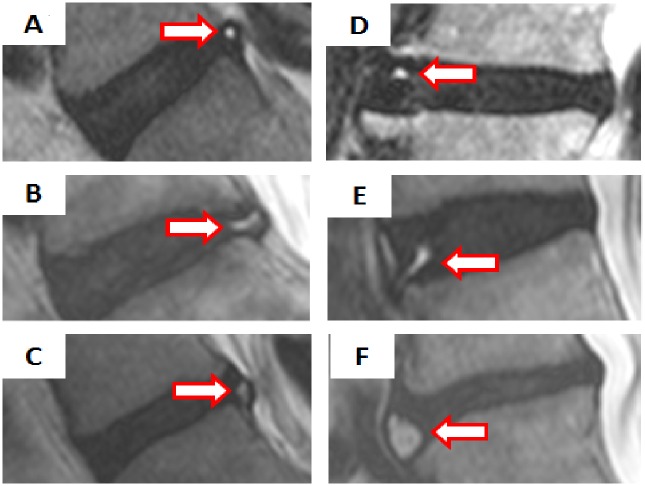
Classification of High Intensity Zones based on morphology and topography. High Intensity Zones (HIZ) were defined as a high intensity signal (white) surrounded by low intensity (black) located in the annulus fibrosus on T2-weighted sagittal MRI. Six types of HIZs were created based on the shape (round type, fissure type, vertical type, rim type, and giant type), and location within the disc (posterior or anterior). The images represent **(A)** posterior round type, **(B)** posterior fissure type, **(C)** posterior vertical type, **(D)** anterior round type, **(E)** anterior rim type, and **(F)** anterior enlarged type.

**Fig 2:**
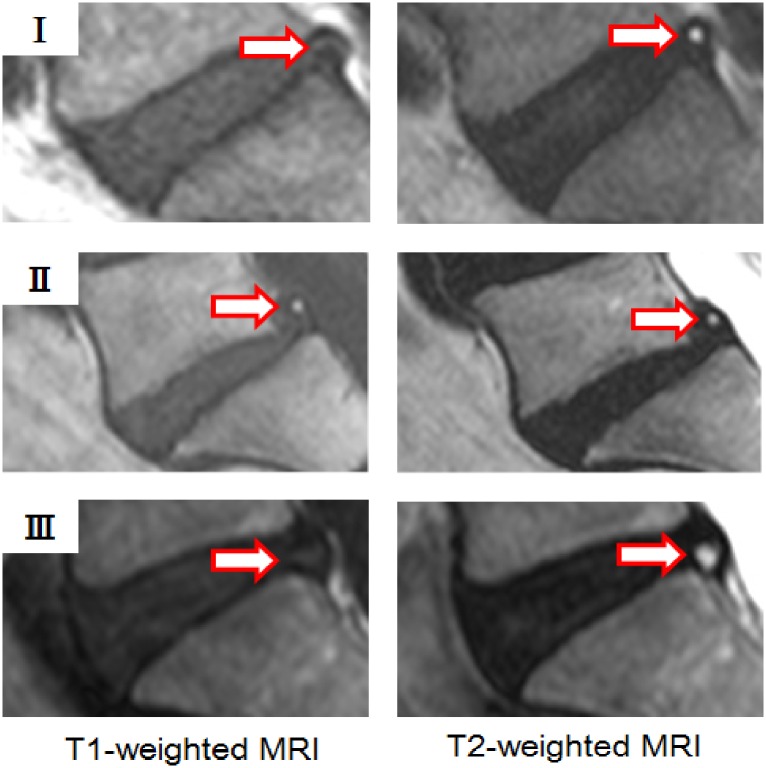
High Intensity Zones based on signal types on T1- and T2- weighted MRI. Three types of High Intensity Zones (HIZ) were created based on the signal type on T1-weighted MRI (low-intensity, high-intensity, and iso-intensity signal) and T2-weighted MRI (high-intensity signal). (I) T1-weighted low-intensity and T2-weighted high-intensity image, (II) T1-weighted high-intensity and T2-weighted high-intensity image, and (III) T1-weighted iso-intensity and T2-weighted high-intensity.

HIZ was defined as a bright white signal located in the substance of the annulus fibrosus, clearly dissociated from the signal of the nucleus pulposus, which was surrounded by a low-intensity (black) signal of the annulus fibrosus and in turn was appreciably brighter than the cerebrospinal fluid signal at the same level on T2W sagittal MR images of L1-S1 [8, 13]. Our novel classification of HIZ was created based on the disc level, shape (round type, fissure type, vertical type, rim type, and enlarge type), and location within disc (posterior or anterior) (Table 1, Fig 1). We also included details regarding the signal type on either T1W MRI (low-intensity, high-intensity, and iso-intensity signal) and T2W MRI (high-intensity signal) (Table 1, Fig 2). The novel classification scheme was developed based on empirical evidence and observational variants as noted between both imaging modalities in the context of HIZ, further agreed to by a panel of experts on spinal phenotyping.

**Table 1:** Assessment of lumbar High Intensity Zones on MRI.

Variables	Definition
***Shape***	
Round	Concentric or oval cavity
Fissure	Parallel and transverse layer to the adjacent endplate
Vertical	Vertical layer to the adjacent endplate
Rim	Oblique radiating layer from the adajacent endplate
Enlarged	Greater concentric area than typical round HIZ
***Horizontal location within disc***	
Posterior	HIZ located in the posterior annulus fibrosus
Anteriror	HIZ located in the anterior annulus fibrosus
***Signal type on T1W and T2W HIZ image***	
T1W low-intensity type of HIZ	Decreased signal than the bone marrow on T1W sagittal MRI
T1W high-intensity type of HIZ	Increased signal than the bone marrow on T1W sagittal MRI
T1W iso-intensity type of HIZ	Same signal than the bone marrow on T1W sagittal MRI

HIZ: high intensity zones, MRI: magnetic resonance imaging, T1W: T1-weighted, T2W: T2-weighted, MRI: magnetic resonance imaging

Sagittal T2W and T1W MRI were used to assess the intervertebral space from L1/L2 to L5/S1. HIZ assessment was performed by a board certified orthopedic surgeon (MT) who was blinded to the background of the subjects. For evaluating intra-observer variability, 20 randomly selected lumbar MRIs were rescored by the same observer (MT) more than 1 month after the first reading, again blinded to the patient details. For inter-observer variability, another 20 MRIs (100 discs) were scored by 2 board certified orthopedic surgeons (MT and HI) using the same classification system. The intra- and inter-observer reliabilities for HIZ on T2W MRI were evaluated by kappa analysis and were 0.92 and 0.84 (p<0.0001, 95% confidence interval (CI): 0.96–1.06), respectively. As for the intensity of HIZ on T1W-MRI, kappa analysis of the intra- and inter-observer reliabilities were 0.90 and 0.82 (p <0.0001, 95% CI: 0.83–0.95). Kappa statistics >0.90 were considered excellent, 0.80–0.90 were considered good, 0.60–0.80 were considered fair, and <0.60 were considered poor [39, 40]. Any disagreements in classification were settled by consensus after the reliability assessments were completed. The final classification of HIZ was agreed upon by both observers and DS.

Other spinal MRI phenotypes, such as DD, disc displacement, Modic changes, and Schmorl’s node (SN) were also assessed by two board certified orthopedic surgeons (MT and RK). DD was classified by grade 4 or 5 on sagittal T2W MRI based on Pfirrmann’s classification [41]. Disc displacement was evaluated as a disc bulge, protrusion, or extrusion. Disc bulge was defined as a disc displacement posteriorly beyond the line of the posterior edges of the adjacent vertebral bodies. Disc protrusion was noted as the nucleus displacement beyond the confines of the annulus fibrosus. Disc extrusion was recognized when the distance between the edges of the disc material beyond the disc space was greater than the distance between the edges of the base of the disc material beyond the disc space [42, 43]. Modic change was defined as diffuse areas of signal change along the endplates, and always parallel to the vertebral end plates on sagittal T1 and T2W images. Modic classification was based on the description originally proposed by Modic *et al* [44] on MRI: Type I was defined as decreased signal intensity on T1W and increased signal intensity on T2W, Type II change was defined as increased signal intensity on both T1W and T2W, and Type III change was defined as decreased signal intensity on both T1W and T2W. Endplate abnormality in any rostral or caudal endplate were assessed as SN defined as a local vertebral endplate defect/abnormality in deviation of the typical concavity or flattened continuous shape of the endplate [30,45]. The intra- and inter-observer reliabilities of these additional MRI phenotypes have been previously reported to be good to excellent [30, 39, 40].

### Statistical analysis

All statistical analyses were performed using JMP version 8 (SAS Institute Japan, Tokyo, Japan). Prevalence of HIZ was examined both per subjects and per disc level. Presence of HIZ was defined as having at least one HIZ in the lumbar region. Moreover, we assessed the prevalence of HIZ regarding shape (round type, fissure type, vertical type, rim type, and enlarge type), location within disc (posterior or anterior), and signal types on T1W MRI of HIZ in the lumbar region and at each affected lumbar disc level, respectively. Pearson χ^2^ test and ANOVA (analysis of variance) with within group Tukey post-hoc tests were used to assess the association between HIZ and no HIZ, between posterior HIZ and anterior HIZ, and among T1W low-, high-, and iso- intensity type of HIZ where applicable. Non-paired student t-test was performed to compare continuous Pfirrmann grade at HIZ affected disc level. The threshold for statistical significance was a p-value less than 0.05.

## Results

There were 814 individuals who underwent lumbar MRI assessment, of which 246 were males (30.2%) and 568 were females (69.8%). The mean age of the subjects was 63.6 years (SD: ±13.1 years). The mean age of males was 63.1 years (SD: ±14.0 years) and the mean age of females was 63.8 years (SD: ±12.7 years). The mean height was 166.8 cm (SD: ±6.7 cm) in males and 153.3 cm (SD: ±6.4 cm) in females. The mean weight was 66.8kg (SD: ±11.0kg) in males and 53.1 kg (SD: ±9.0 kg) in females. In addition, the mean body mass index (BMI) was 24.0 kg/m^2^ (SD: ±3.6 kg/m^2^) in males and 22.6 kg/m^2^ (SD: ±3.6 kg/m^2^) in females.

### Prevalence of HIZ

**Fig 3:**
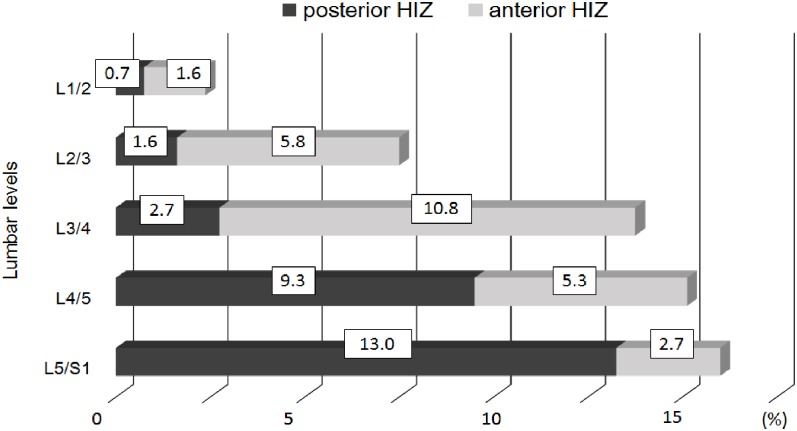
Bar chart showing the overall percent prevalence of anterior and posterior High Intensity Zones per lumbar level. Posterior HIZ was most common at L5/S1 followed by L4/5. Alternatively, anterior HIZ had the highest prevalence at L3/4 followed by L2/3.

HIZ were noted in 38.0% (n = 309) of all participants, and within these subjects the prevalence of posterior HIZ, anterior HIZ, and both posterior/anterior HIZ in the overall lumbar spine were 47.3% (n = 146), 42.4% (n = 131), and 10.4% (n = 32), respectively. Of the 309 HIZ subjects, 26.0% had single HIZ (n = 212), 8.6% had 2 HIZs (n = 70), 2.7% had 3 HIZs (n = 22) and 6.1% had 4 HIZs (n = 5). Of these subjects, involved discs only had a single HIZ. In addition, of the 97 multilevel HIZ subjects, 71.1% had consecutive level HIZs (n = 69) and 26.9% had skipped level HIZs (n = 28). The overall percentage prevalence of posterior and anterior HIZ per lumbar levels is illustrated in Fig 3. Posterior HIZ was most common at L5/S1 followed by L4/5. Alternatively, anterior HIZ had the highest prevalence at L3/4 followed by L2/3. As such, region-specific variations between upper (L1-L4) and lower (L4-S1) lumbar spine HIZ were noted.

### Morphology and topography of HIZ

Table 2 illustrated the morphological distributions of HIZs of the lumbar discs. Round type HIZ (Fig 1A and 1D) were most common in both posterior and anterior discs. Furthermore, round type HIZ in the posterior disc was more common at L4/L5 and L5/S1, whereas round type HIZ in the anterior disc was most common at L2/L3 and L3/L4. Fissure type and vertical type HIZ in the posterior disc (Fig 1B and 1C) was most common at L5/S1 and L4/L5. Rim type and enlarged type HIZ in the anterior disc (Fig 1E and 1F) were most common at L3/L4 and L4/L5. In addition, of the 309 subjects with HIZ, 222 (71.8%) had T1W iso-intensity type of HIZ (Fig 2 type III), followed by 66 (21.4%) with T1W high-intensity type of HIZ (Fig 2 type II) and 21 (6.8%) with T1W low-intensity type of HIZ (Fig 2 type I). As for disc level, T1W iso-intensity type of HIZ was most common at L4/L5 (11.5%, n = 94) followed by L5/S1 (11.4%, n = 93), T1W high-intensity type of HIZ was the highest at L5/S1 (3.7%, n = 30) followed by L4/L5 (2.9%, n = 24), and T1W low-intensity type of HIZ was the highest at L4/L5 (1.0%, n = 8) followed by L3/L4 (0.9%, n = 7).

**Table 2:** Distribution of shapes of High Intensity Zones at lumbar levels (n: 814 subjects).

Disc level	Posterior round, n (%)	Posterior fissure, n (%)	Posterior vertical, n (%)	Anterior round, n (%)	Anterior rim, n (%)	Anterior enlarged, n (%)
**L1/L2**	6 (4.0)	0 (0)	0 (0)	11 (7.5)	2 (3.7)	0 (0)
**L2/L3**	9 (6.0)	0 (0)	4 (6.2)	42 (28.8)	5 (9.3)	0 (0)
**L3/L4**	16 (10.6)	0 (0)	6 (9.2)	61 (41.8)	21 (38.9)	6 (40.0)
**L4/L5**	49 (32.5)	3 (42.9)	24 (36.9)	20 (13.7)	17 (30.9)	8 (53.3)
**L5/S1**	71 (47.0)	4 (57.1)	31 (47.7)	12 (8.2)	9 (16.4)	1 (6.7)
**Total**	151(100)	7 (100)	65 (100)	146 (100)	54 (100)	15 (100)

Note, every disc level from L1/L2 to L5/S1 has been individually evaluated.

### Association of other spinal MRI phenotypes

As Table 3 illustrates the presence of HIZ was a clear determinant whether that disc level had other spinal MRI phenotypes or not. Disc levels with HIZ had significantly more disc bulges/protrusions (37.9% vs 29.3%, p<0.01) and DD (median 3.8, SD: ± 0.7 vs. 3.7, SD: ± 0.7, p<0.001), but not extrusions (1.1% vs 1.3%, p = 0.97). Modic type II change was significantly associated with HIZ at the affected vertebral body adjacent to the end plate (27.9% vs. 21.4%, p<0.01).

**Table 3:** Associated variables with High Intensity Zones at affected lumbar levels.

Variables	HIZ	No HIZ	p- value	Posterior HIZ	Anterior HIZ	p- value	T1W low-intensity type of HIZ	T1W high-intensity type of HIZ	T1W iso-intensity type of HIZ	p- value
**Total discs; 4070**	438	3632		223	215		22	80	339	
***HIZ affected disc level***										
**Disc bulges/protrusions, n (%)**	166 (37.9)	1065 (29.3)	<0.01	96 (43.0)	70 (32.6)	<0.001	9 (41.0)	36 (45.0)	123 (36.3)	<0.01
**Extrusions, n (%)**	5 (1.1)	48 (1.3)	0.97	4 (1.8)	1(0.5)	0.33	0 (0)	2 (2.6)	3 (0.9)	0.52
**Disc degeneration (mean ±SD)**	3.8± 0.7	3.7±0.7	<0.001	3.8±0.7	3.6 ± 0.7	<0.001	3.7±0.8	3.9±0.7	3.8±0.6	<0.01
***HIZ affected vertebral body adjacent to the end plate (total endplates;4070)***										
**Modic type I, n (%)**	24 (5.5)	176 (4.9)	0.29	13 (5.8)	11 (5.1)	0.32	0 (0)	7 (8.8)	17 (5.0)	0.18
**Modic type II, n (%)**	122 (27.9)	779 (21.4)	<0.01	75 (33.6)	47 (21.9)	<0.001	7 (31.8)	22 (27.5)	94 (27.7)	<0.05
**Modic type III, n (%)**	14 (3.2)	88 (2.4)	0.18	13 (5.8)	1 (0.5)	<0.0001	0 (0)	3 (3.8)	11 (3.2)	0.4
**Schmorl's node, n (%)**	101 (23.1)	707 (19.5)	0.075	50 (22.4)	51 (23.7)	0.19	3 (13.6)	21 (26.3)	74 (21.8)	0.75

Pearson χ² test and ANOVA (analysis of variance) with within group Tukey post-hoc tests were used to assess the association between HIZ and no HIZ, between posterior HIZ and anterior HIZ, and among T1W low-, high-, and iso- intensity type of HIZ where applicable. Non-paired student t-test was performed to compare continuous Pfirrmann grade at HIZ affected disc level. High-intensity zones (HIZ), T1W: T1-weighted, SD: standard deviation, %: percentage, n: number of subjects.

Posterior HIZ had more bulges/protrusions (43.0% vs. 32.6%, p<0.001) and DD (median: 3.8, SD: ± 0.7 vs. 3.6, SD: ± 0.7, p<0.001) than anterior HIZ. Modic type II change was more significantly associated with posterior HIZ at each affected vertebral body (33.6% vs. 21.9%, p<0.001), Modic type III change was in comparison more significantly associated with posterior HIZ (5.8% vs. 0.5%, p<0.0001).

When comparing T1W low-intensity, T1W high-intensity and T1W iso-intensity types of HIZ, T1W low-intensity and high- intensity types of HIZ had more bulges/protrusions as compared with T1W iso-intensity type of HIZ (41.0% vs. 46.8% vs. 36.0% p<0.01) and DD (median: high 3.7, SD: ± 0.8 vs. low 3.9, SD: ± 0.7, vs iso 3.8, SD: ± 0.6, p<0.01). Modic type II change was significantly associated with T1W low-intensity type of HIZ than T1W iso- and high intensity types of HIZ (31.8% vs. 28.6% vs. 27.4%, p<0.05). (Table 3)

## Discussion

Our large-scale population-based study presents a novel classification scheme of HIZ based upon evaluation of the morphology, topography, and the relationship of T1W and T2W MRI signal changes of HIZ. This classification is more precise and comprehensive than what has been traditionally reported and can be utilized for any future analysis regarding phenotype association and clinical relevance. Furthermore, to our knowledge, this study is also the first to address HIZ and their association of the other MRI spinal phenotypes based on both T1W and T2W MRI.

Since the original description of the HIZ on T2W sagittal MRI in 1992 [8], the prevalence of HIZ has varied greatly between reported studies in spite of the subjects with or without LBP. The prevalence of posterior HIZ was reported to be from 28.6% to 59% in symptomatic patients [8–11, 13] as compared to 3.2% to 24% in asymptomatic subjects [13–16]. Our large-scale population study in comparison showed that the prevalence of posterior HIZ was 21.9% (179/814 subjects). We also found posterior HIZ to be most common at L5/S1 (13.0%) followed by L4/L5 (9.3%), which was supported by a few studies [8, 10, 15]. However, we also report anterior HIZ to commonly occur at L3/L4 (10.8%) followed by L2/L3 (5.8%). This finding underscores the fact that region-specific variations of HIZ exists within the lumbar spine, with distinction between the upper (i.e. L1-L4) and lower (i.e. L4-S1) lumbar discs. Recent studies have noted more of a developmental origin or predisposition of upper lumbar segment phenotypes [46]. Nonetheless, the fact that HIZ is frequently found to be at the anterior of the disc is contrary to the traditional belief that HIZ must be posterior [8–18]. Hence, the lack of standardization for classifying HIZ for including anterior HIZ may be a likely reason for the discrepancies in the current literature regarding the reported prevalence.

Provocation discography has been utilized for assessment of annular tears and LBP [17, 18, 47, 48]. However, discography remains controversial due to the associated risks. For example, the procedure is invasive and complications include infection (epidural abscess, discitis), neurological injury, and possible contrast medium reaction [48, 49]. There is also the possibility of increased progression of DD and herniation after the examination [48, 49]. Therefore, to allow for future non-invasive HIZ research, Yu *et al* [17] reported the sensitivity of HIZ to diagnose annular tears on MRI with discography and cadavers and concluded that HIZ demonstrated some radial tears of annulus in 1989. With our more thorough MRI study with advanced sequences and imaging technique, our findings and classification of morphological/topographical variants of HIZ will further enhance our understanding of the pathology of intervertebral disc disorder. This allows us to have a more sensitive and non-invasive method of identifying symptomatic disc levels, predicting disc changes, and potential use for patient selection for disc regenerative therapies. This also has potential to be a marker for identifying patients at risk for adjacent segment degeneration/disease in relation to a fusion or arthroplasty procedure.

Various proposals have been put forward to explain the discrepancy between the presence of HIZs in asymptomatic and symptomatic individuals [8–16]. Six years after the initial paper [8], Bogduk postulated that annular tears may be present in asymptomatic subjects as low-intensity zones on T2W MRI, and these may become painful and assume a brighter signal to become an “activated” HIZ [50]. Indeed, the present study is in concurrence as we did not find low intensity zones on T2W MRI. Bogduk also reported an inability to detect HIZ on T1W MRI [8]. However, this is disputed in our study as we observed variable intensity types of HIZ on T1W MRI. Hence, we believe that coupling of T2W and T1W MRI sequences is necessary to define the HIZ phenotype. HIZ has been defined as collections of mucoid fluid within the annulus tear and thus have a bright signal on T2W MRI in the pathological studies [8, 10, 14]. However, HIZ may also change and represent a reflection in the pathological process, which may convert from one type to another, for example, neovascularization of annulus, a healing annular tear, and fluid or mucoid material filled in the inflamed annular tear. These processes may express as different signals on T1W MRI.

We found in this study significant associations between the presence of HIZ and DD, disc bulge/protrusion and Modic type II changes at all affected levels. These results support the view that degenerative findings and HIZ co-exist. Some investigators have suggested that HIZ was a part of the degenerative process as HIZs occurred in association with degenerative changes within the disc [9,10,13], whereas others disagreed [14]. This discrepancy is partly explained by the sample population, presence or not of symptoms and how clinical parameters are defined, small sample size, and/or insufficient statistical analyses. However this large-scale, population-based study identified a strong association between HIZ with DD and disc bulge/protrusion. We also found that Modic Type II changes were more associated with the presence of HIZ, especially posterior HIZ, T1W low-intensity type of HIZ. In addition, Modic type III change was more associated with posterior HIZ than anterior HIZ. These relationships are understandable and can be attributed to the altered biomechanics associated with endplate failure caused by HIZ or as a reverse causality of Modic changes leading to HIZ. Furthermore, Schmidt *et al* [51] showed that HIZ was associated with instability of the intervertebral disc which caused fluid to move through annular tear into the outer annulus [15]. Subsequently, the unstable motion of intervertebral disc increased the stress and strain at adjacent disc segments, leading to Modic change [52]. Thus, HIZ and its sub-phenotypes may have potential as imaging biomarkers to identify those patients at risk for DD, instability of disc, and adjacent segment degeneration/disease. In general, studies have noted that Modic changes are highly associated with LBP; however, different degrees of pain severity and disability may exist [4–6]. There are also subjects with Modic changes and no HIZ. As such, being able to identify clinically relevant HIZ associated with Modic changes may shed additional light into identifying more problematic disc levels.

These results of our study may be influenced by the high age groups of our cohort (mean age over 60 years); thus, additional study is necessary to further assess HIZ among different age strata. Moreover, as with all population-based studies, there may be an effect of ethnic variability that should be addressed in future studies [53]. In addition, due to the availability of scanning units at the initiation of our study, we utilized a mobile 1.5 T MRI unit to facilitate the assessment of our subjects. Although a higher field strength, such as 3T MRI, may theoretically have a higher sensitivity in detecting specific HIZs; there have been no studies that have addressed such a concern to date to gauge the extent of the variation and it was not an aim of our current study. However, it is also important to consider that all subjects in our current study were assessed via the 1.5T MRI, representing a consistency in assessment. Our work raises awareness of the variation of HIZs that may exist in the lumbar spine and we hope will form the much needed foundation for future studies to explore upon this research platform to a much greater extent. Finally, the current study did not address an association of HIZ with LBP due to the limited pain profile assessment available in the cohort. Importantly, the strength of the present study is the size of the study population and the novel in-depth multi-parametric phenotype profiling on MRI that could serve as the basis for future HIZ study and phenotype standardization in the future. Such a foundation can then be utilized to assess more in-depth clinical relevance and utility.

## Conclusions

This is the first large-scale, population-based study to systematically assess the epidemiology of HIZ on 1.5T MRI and report upon a novel classification of this phenotype in the lumbar spine. In addition, this study is also the first to utilize a multi-parametric imaging approach to assess the different variants of HIZ by the use of T2W and T1W MRI. Hence, with such alternative imaging in mind, it may be appropriate in the future to not refer to the HIZ phenotype as representing “high” intensity zones but rather “intensity zones”. Such a nomenclature may be more apropos given that some HIZ on T1W MRI are not “high” intensity. Although HIZ is frequently found to be posterior, as traditionally believed, they do occur anteriorly in the disc, and numerous morphological variants exist that are disc-level and region-specific, and distinguishable via a multi-parametric imaging approach. Furthermore, HIZ are highly associated with specific MRI spinal phenotypes, such as DD, disc bulges/protrusions, and Modic changes. In an age whereby various “omics” approaches and large data set cohorts are becoming more commonplace, a standardized phenotype classification of HIZ is imperative. Such a scheme can be further utilized to assess the clinical profile of patients, identify problematic discs, prognosticate outcomes and help tailor specific spine treatments. Additional, large-scale, comparative prospective studies are needed to further validate our findings and address their clinical impact.

## Abbreviations

HIZ: high intensity zone
DD: disc degeneration
MRI: magnetic resonance imaging
ROAD: Research on Osteoarthritis/Osteoporosis Against Disability
